# Prolonged Hypercalcemia-Induced Psychosis

**DOI:** 10.1155/2020/6954036

**Published:** 2020-02-01

**Authors:** Lauren Nagy, Pratheek Mangini, Caitlin Schroen, Rehan Aziz, Anthony Tobia

**Affiliations:** ^1^Department of Psychiatry, Rutgers Robert Wood Johnson Medical School, Piscataway, NJ, USA; ^2^Rutgers Robert Wood Johnson Medical School, Piscataway, NJ, USA

## Abstract

Hypercalcemia is known to cause neuropsychiatric dysfunction including mood and cognitive changes and rarely, acute psychosis. High calcium levels can be a catalyst for neuronal demise, possibly due to glutaminergic excitotoxicity and dopaminergic and serotonergic dysfunction. While restoration of normal calcium levels or removal of a parathyroid adenoma has been shown to rapidly resolve neuropsychiatric symptoms, there have been rare reported cases of primary hyperparathyroid-related hypercalcemia with persistent symptoms of psychosis. In this case report, we will describe a patient with no past psychiatric history presenting with a protracted course of delirium and psychosis after a removal of a parathyroid adenoma which had caused prolonged exposure to hypercalcemia. The patient's psychosis was unresponsive to psychotropic medication and required inpatient psychiatric care after medical clearance. Per medical records, before the patient was ultimately lost to follow-up, she continued to suffer from psychotic symptoms for at least 8 months. We will discuss the patient's unusual hospital course and management and offer suggestions for future study.

## 1. Introduction

Hypercalcemia is known to cause neuropsychiatric dysfunction. In mild cases, patients may present with anxiety, depression, and cognitive changes, while altered mental status, psychosis, confusion, lethargy, and coma hallmark severe hypercalcemia [1]. Several cases of hypercalcemia-related delirium have been reported in the literature, often secondary to hyperparathyroidism, parathyroid adenoma, lithium toxicity, and cancer [2–5]. These cases describe patients presenting with sudden alteration in mental status accompanied by auditory hallucinations, paranoia, and persecutory delusions [2–5]. The mechanism of hypercalcemia-induced psychosis remains poorly understood but may be explained by alterations in central nervous system (CNS) monoamine levels as well as glutamate-mediated excitotoxicity, as detailed below.

First, dopaminergic dysfunction has long been implicated in psychosis [6]. In hypercalcemia-induced psychosis due to parathyroid adenoma, reduced levels of dopamine, serotonin, and norepinephrine have been found in the cerebrospinal fluid (CSF) [7]. Surgical resection of the adenoma usually corrects these abnormalities and correlates with clinical improvement, particularly affective symptoms [7]. This implies that hypercalcemia-induced psychiatric disturbances may be mediated by dopaminergic and serotonergic dysfunction in the CNS.

Glutaminergic excitotoxicity via N-methyl-D-aspartate (NMDA) receptors is another possible etiology for hypercalcemia-induced psychosis. Glutamate acts as a ligand to the NMDA receptor, allowing calcium influx from the extracellular space [8, 9]. Activation of the NMDA receptor is associated with long-term potentiation and is integral to neuroplasticity [9]. Glutamate neurotoxicity is caused by increased intracellular calcium influx via NMDA receptor activation [8]. Blocking NMDA receptors and removing calcium from the extracellular space reduces excitotoxicity [10]. Increased calcium levels contribute to neuronal demise by facilitating increased mitochondrial permeability—leading to mitochondrial rupture, cell dysfunction, and death [8]. Neuronal demise may also be caused by GABA receptor overactivation in the context of cerebral ischemia [11].

Given that high calcium levels can be a catalyst for neuronal demise, excitotoxicity, and alteration of key neurotransmitters, it is unsurprising that hypercalcemia causes altered mental status and psychotic features. As expected, restoration of normocalcemia has been shown in the majority of cases to rapidly resolve neuropsychiatric symptoms, as early as 1 week after surgery [12, 13]. However, there are rare reported cases of primary hyperparathyroid- (PHPT-) related hypercalcemia with persistent psychosis. One published example is that of a patient with persistent paranoia and violent behavior for 2-3 months following parathyroidectomy, requiring psychiatric care for 6 months [14].

In this case report, we will describe a patient with a protracted course of delirium and psychosis after removal of a parathyroid adenoma which caused prolonged hypercalcemia. We will examine the patient's hospital course and management and offer suggestions for future study.

## 2. Case Report

The patient is a 58-year-old Haitian Creole-speaking female with no significant past medical, psychiatric, or substance use history who was brought to her primary care doctor by her family following three weeks of headache and change in mental status, including paranoia and auditory hallucinations. The patient's family reported that she was in her usual state of health when she suddenly began to exhibit “bizarre behavior,” hiding in closets out of fear that people were going to “beat her up and kill [her],” and hearing voices of these threatening people. During this time, the patient also had symptoms of headache, joint pain, and constipation. Bloodwork completed by her family physician found her calcium to be 14.4 (normal 8.5-10.2) mg/dL, and she was subsequently transferred and admitted to the medical unit at our hospital.

After admission, the patient was found to have a total calcium of 14.4 mg/dL, ionized calcium of 7.2 (normal 4.64 to 5.28) mg/dL, parathyroid hormone (PTH) level of 759 (normal 10-65) pg/dL, albumin of 4 (normal 3.5-5.5) g/dL, and phosphorous of 2 (normal 2.5-4.5) mg/dL, concerning for a diagnosis of PHPT. No other medical cause for delirium was found; the patient had consistently stable vitals and negative workup for sepsis, HIV, syphilis, and toxicological causes. The patient was treated with IV fluids and calcitonin. Ultrasound of the neck found multiple nodules on the right thyroid gland consistent with parathyroid adenoma, confirming the diagnosis of primary hyperparathyroidism. The patient was managed medically until her surgery could take place, with calcium levels fluctuating between 10 mg/dL and 12.9 mg/dL. As workup was completed, our Psychiatry Consultation and Liaison (C/L) team was consulted to assess her mental status and recommend and manage pharmacotherapy for delirium. The patient's primary language was Haitian Creole, and as such, interviews were conducted utilizing a phone-translation service, with information often verified by her bilingual family members. As aspects of the Mini-Mental State Examination (MMSE) are based on ability to read and write in English, some components of the MMSE were not possible to assess using phone-translation and were excluded from scoring, as noted below.

Our initial evaluation was on hospital day 2, with calcium now corrected with medical intervention to 10.2 mg/dL. Our exam was significant for somnolence, unchanged auditory hallucinations and persecutory delusions, poverty of speech, fluctuation in attention, and poor concentration. The patient was oriented only to person and knew that she was in a hospital. She was guarded with the interviewer, expressing fear that her clinical team would reveal her location to the “people trying to get [her].” During her stay, she periodically endorsed suicidal ideation, with two attempts at self-asphyxiation with an IV cord. She also was often found by night staff hiding in her closet from “people coming to get [her].” She was diagnosed with delirium due to hypercalcemia, mixed-subtype.

Our C/L team recommended one-to-one (1 : 1) observation, frequent reorientation, and limiting the use of tethers (e.g., catheters), as well as other environmental maneuvers as part of the hospital's delirium protocol. Her psychosis was treated with olanzapine titrated to 15 mg daily, gabapentin 300 mg daily, and haloperidol 2 mg IM every four hours for agitation that impeded essential medical care. These interventions yielded no significant change in mental status or perceptual disturbances.

Six days before right hemithyroidectomy, the patient was evaluated by the C/L team with calcium level of 10.4 mg/dL, ionized calcium of 5.2 mg/dL, albumin of 3.5 g/dL, and PTH of 8 pg/dL. Her MMSE was scored 11/22, with some questions deferred due to language barrier. Patient lost points for date, season, and declined to answer location questions or copy a drawing of intersecting pentagons. There was no change in the patient's mental status exam at this time; she continued to have auditory hallucinations of voices stating they were going to “tie a rope around [her] neck.” She also expressed persecutory delusions that these people came to the hospital and tried to enter her room the night before.

Six days postsurgery, with calcium level of 7.7 mg/dL, albumin of 4.1 g/dL, and PTH of 91 pg/mL, the patient was found to have continued somnolence, fluctuating attention, disorientation to place and time, and a persecutory delusion that she would be “arrested.” She denied hallucinations or suicidal ideation at this time. She was found to have an MMSE score of 4/18, losing points for all time and place questions, attention, and 2/3 delayed word recall, again with some questions deferred due to language barrier.

Eleven days postsurgery, with calcium level of 8.8 mg/dL, albumin of 4 g/dL, and PTH of 63 pg/mL, the patient was found to have improved alertness and was oriented to person, hospital, day, month, and year but not to city or state, despite continued treatment with olanzapine, gabapentin, and haloperidol. She denied hallucinations but her persecutory delusion of being “arrested” remained, and the patient expressed feeling unsafe in the hospital. The patient exhibited echolalia as well as blunted affect. She continued to have some impairments but showed marked improvement, with MMSE score of 17/30. Points were lost for date, town, county, hospital floor, concentration, phrase repetition, writing a sentence, copying a picture, and 2/3 words remembered on delayed recall.

The patient was discharged on postoperative day 13, hospital day 29, to an outside inpatient psychiatric unit for management of continued psychosis with no further improvement of her MMSE. While the patient was no longer in our direct care, she sought sporadic treatment through our hospital system following discharge and some of her course is known. The patient was discharged after 6 days from inpatient psychiatric care with a medication regimen including olanzapine 10 mg and gabapentin 600 mg three times daily and attended one appointment with our outpatient psychiatry office. The patient was lost to follow-up for 4 months until she presented to our hospital again for symptoms of disorientation, drowsiness, and slowed gait. She was then admitted for a second time, 5 months after initial presentation, and was diagnosed with extrapyramidal symptoms from antipsychotic use. She was discharged the next day with symptom resolution on 20 mg propranolol daily. Her olanzapine was discontinued and an appointment with her psychiatrist was made. Unfortunately, our records show that the patient next sought out care again 3 months later, when her family called crisis outreach for bizarre behavior, auditory hallucinations, and persecutory delusions. She was admitted to a nonaffiliate inpatient psychiatric service 8 months after initial presentation, and her current clinical status is unknown.

## 3. Discussion

Our C/L team was consulted by the primary medical team to manage this patient's psychosis, severe agitation, and suicidal gestures while medical and surgical management of her hypercalcemia was addressed. Based on previous studies, it was our team's expectation that upon restoration of normocalcemia, we would also observe a rapid resolution of her psychosis. While it has been established in the literature that some cases of new-onset psychosis secondary to hypercalcemia from parathyroid adenoma may have prolonged courses, there is little discussion as to the appropriate medical management of these patients with persistent symptoms weeks or months after surgery. This is further compounded by our limited understanding of the mechanism of hypercalcemia-induced psychosis.

Our approach used olanzapine as the primary agent to manage her psychotic symptoms as it modulates activity at both serotonin and dopamine receptors; prior studies have proposed altered metabolism of monoamines as the mechanism of psychosis in PHPT [7, 15]. A case report of primary parathyroid adenoma in a pregnant patient showed successful reduction of psychotic symptoms with olanzapine for 5 weeks until the patient was cleared for surgical resection in the second trimester [16]. Additionally, olanzapine and other atypical antipsychotics have demonstrated efficacy in the treatment of delirium symptoms [17]. Given these findings, olanzapine was selected to be the primary agent in reducing psychotic symptoms with haloperidol to be used as needed for agitation.

Unfortunately, our patient did not improve significantly on olanzapine, continuing to exhibit paranoia and auditory hallucinations. It is likely that there was a concomitant mechanism, in addition to potential altered monoamine metabolism, that led to persistent psychosis in our patient with long-standing hypercalcemia. As such, it is possible that antagonism of dopamine and serotonin receptors could have further exacerbated the pathologic state of increased metabolism of these neurotransmitters. It is also possible that low levels of monoamine metabolites in the CSF may be responsible; studies have found that schizophrenic patients with low levels of dopamine metabolites in the CSF, compared to those with normal levels, do not respond well to neuroleptics [18].

Another proposed mechanism for psychosis in PHPT is hypercalcemia-induced NMDA excitotoxicity. To combat this effect, gabapentin was added as adjuvant therapy after several days of poor response to olanzapine. One study has shown that gabapentin can provide a neuroprotective effect against excitotoxicity in rat hippocampal-CA1 neurons by inhibiting voltage-gated calcium currents of NMDA receptors [19]. It was hypothesized by our team that gabapentin would reduce the neurotoxic effect of hypercalcemia on NMDA receptor activity; however, the addition of this agent provided little clinical improvement, though the dosing may have been inadequate.

Finally, additional consideration should be given to the cultural challenges presented by this patient's background. While unlikely given that Haitian-born family members identified her psychotic symptoms as new in this case, it should be acknowledged that the cultural understanding of mental illness in Haiti may have influenced perception of any earlier psychotic symptoms in our patient while she lived there. Research indicates that families in Haiti may be reluctant to acknowledge psychotic illness in a relative due to significant stigma, and those affected are less likely to seek out traditional psychiatric care due to a scarcity of mental health practitioners and the prevalence of traditional or community healing practices for physical and mental illness [20]. In discussion with the patient's family, it was our mutual understanding that the patient had not required previous care, psychiatric or traditional, until her presentation in this case.

## 4. Conclusion

Our case highlights the challenge of medically managing a patient with persistent symptoms of psychosis and agitation despite surgical and medical intervention for hypercalcemia. Second-generation neuroleptics such as olanzapine as well as the use of anticonvulsants such as gabapentin proved to be modestly effective in reducing our patient's neuropsychiatric symptoms. It is possible that our patient proved resistant to medical management as she had presented with 3 weeks of neuropsychiatric symptoms prior to admission, implying an even longer course of clinically silent hypercalcemia, possibly leading to irreversible neuronal damage that resulted in at least 8 months of psychotic symptoms.

Future studies could evaluate the role of various neuroprotective agents on the NMDA receptor. Memantine is one such proposed agent; as an “uncompetitive” antagonist of the NMDA receptor, it is distinct from a competitive antagonist. Uncompetitive antagonists selectively inhibit overactivated receptors and prevent excessive inhibition from traditional NMDA receptor antagonists [19]. While not a common phenomenon, patients with persistent psychosis secondary to parathyroid adenoma present high burdens in clinical care often requiring 1 : 1 observation, concerns for harm to self and others, and difficulty assessing mental status through interview.

Three RCTs have assessed the neurocognitive benefit of parathyroidectomy and produced variable responses; however, surgery is still strongly recommended by the American Association of Endocrine Surgeons for patients unable to comply with observation protocols [21]. While most cases resolve shortly after surgical intervention, inpatient psychiatric care for persistent cases should be considered in hypercalcemia-induced psychosis based on the patient's symptomatology. Further studies identifying effective medical management strategies will be vital in ameliorating these complex clinical courses.
